# ATP-induced P2X Receptor-Dependent Large Pore Formation: How Much Do We Know?

**DOI:** 10.3389/fphar.2016.00005

**Published:** 2016-01-29

**Authors:** Linyu Wei, Emily Caseley, Dongliang Li, Lin-Hua Jiang

**Affiliations:** ^1^Department of Physiology and Neurobiology, Xinxiang Medical UniversityXinxiang, China; ^2^Faculty of Biological Sciences, School of Biomedical Sciences, University of LeedsLeeds, UK

**Keywords:** ATP, P2X receptor, large pore formation, ion-permeating pathway

P2X receptors are a group of homo/hetero-trimeric membrane protein complexes with an integral ion channel that opens upon extracellular adenosine triphosphate (ATP) binding (North, 2002; Khakh and North, 2006). There are seven P2X subunits (P2X1-P2X7), all having a membrane topology of cytosolic N- and C-termini, and two transmembrane segments (TM1 and TM2) connected by a large extracellular domain (Figure 1A; Jiang L.-H. et al., 2013). During application of ATP for a few seconds, P2X receptors function as classical ligand-gated ion channels selectively permeable to small physiological cations such as Ca^2+^, Na^+^, and K^+^, with the exception of the human P2X5 receptor which exhibits significant Cl^−^ permeability (Bo et al., 2003). Site-directed mutagenesis and functional studies of mammalian P2X receptors, in addition to the determination of the crystal structures of zebrafish P2X4 receptors in the apo, closed state and ATP-bound, open state, have defined the structural basis for ATP binding, ion permeation and channel gating (Kawate et al., 2009; Browne et al., 2010; Hattori and Gouaux, 2012; Jiang L.-H. et al., 2013; Jiang R. et al., 2013). Three ATP-binding pockets are located at the subunit interfaces (Figure 1B), each consisting of highly conserved residues from two adjacent subunits. Occupation of these sites by ATP or its synthetic analog agonists induces conformational changes of the extracellular domain which open the ion-permeating pathway formed by three TM2s (Figures 1C,D). The narrowest part of the ion-permeating pathway or the physical gate is provided by A347 and L351 in the crystal structures of zebrafish P2X4 receptor (Hattori and Gouaux, 2012) or the corresponding residues S342 and L346 in the structural models of rat and human P2X7 receptors (Figure 1D) (Bradley et al., 2011; Browne et al., 2013; Jiang L.-H. et al., 2013).

**Figure 1:**
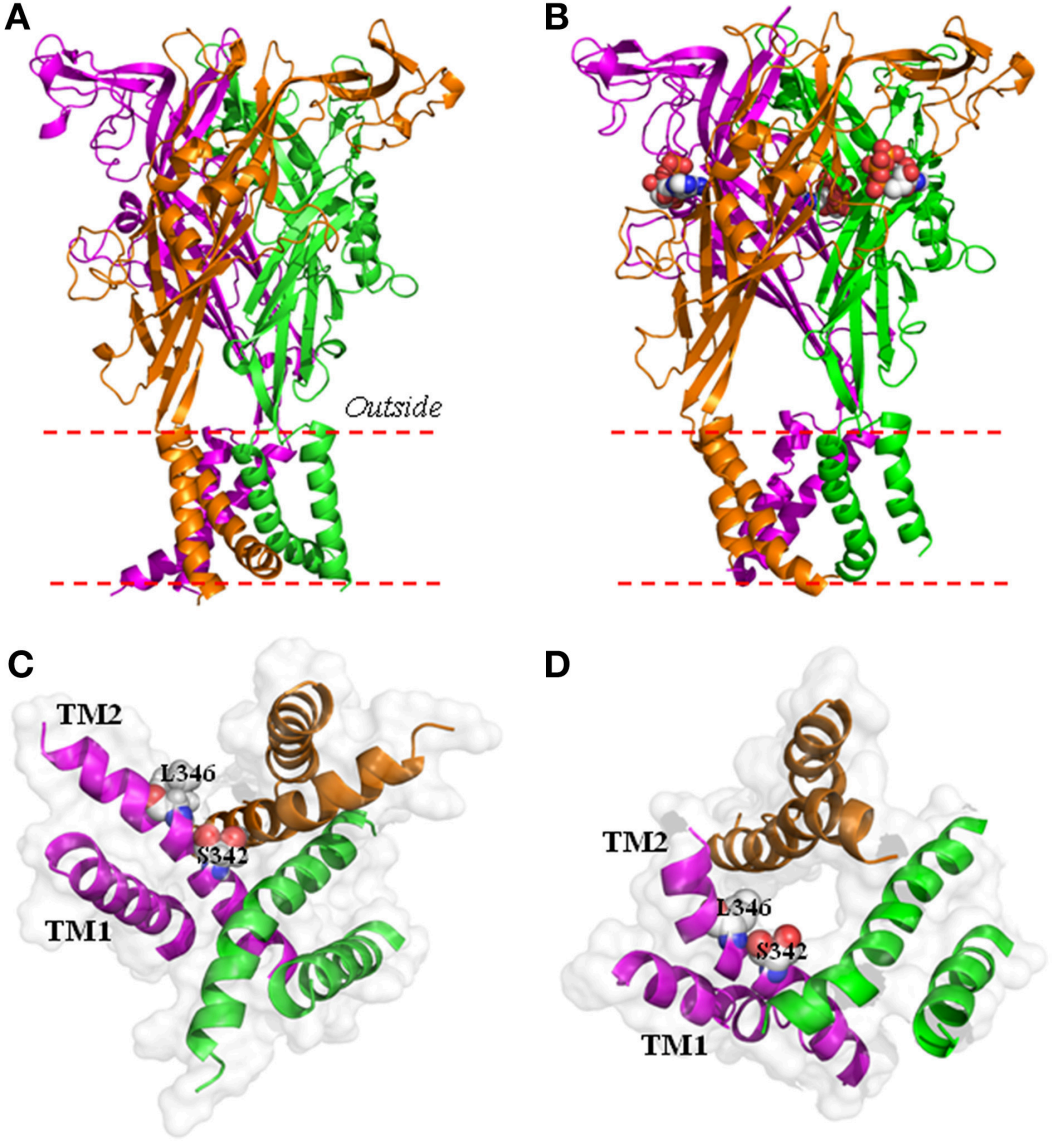
**Structural models of the human P2X7 ion channel in the closed and open state**. **(A,B)** The structural models of the trimeric human P2X7 receptor in the closed **(A)** and open states **(B)**, viewed parallel to the plasma membrane. They are generated based on the crystal structures of zebrafish P2X4 receptor (4DW0 and 4DW1 respectively). Each subunit is shown in a different color. Three ATP molecules shown in space filling representation bind to the three inter-subunit interfaces **(B)**. **(C,D)** The transmembrane ion-permeating pathway in the structural models of the human P2X7 receptor in the closed **(C)** and open states **(D)**, viewed from the intracellular side of the membrane. The three TM1 helices are located at the periphery and the three TM2 helices in the center form the ion-conducting pathway. Ser342 and Leu346 from each of the three subunits provide the narrow part of the ion-conducting pathway in the open state **(D)**.

It is well known that extended application of ATP to activate the P2X receptors for tens of seconds or minutes induces a remarkable increase in membrane permeability to large molecules of up to 900 Daltons, a phenotype often referred to as formation of large pores. This was originally documented in immune cells about three decades ago; ATP permeablized cell membranes to nucleotides (Cockcroft and Gomperts, 1979) and the cationic fluorescent dye ethidium in mast cells (Gomperts, 1983), or the anionic organic dyes lucifer yellow and carboxyfluoresceine in mast cells and macrophages (Bennett et al., 1981; Steinberg and Silverstein, 1987). These immune cells express the formerly named P2Z receptor, which is now known as P2X7 receptor, and heterologous expression of P2X7 receptors conferred ATP-induced large pore formation (Surprenant et al., 1996). Such large pore formation has also been observed during sustained activation of other P2X receptors including P2X2, P2X2/3, P2X2/5, and P2X4 receptors (Khakh et al., 1999; Virginio et al., 1999a,b; Compan et al., 2012). Substantial efforts have been devoted to understanding P2X receptor-dependent large pore formation, but it has been difficult to interpret in a unified mechanism all results from studies examining different receptors in different cell types with different receptor expression levels. Two distinctive mechanisms or hypotheses have been proposed (North, 2002; Pelegrín, 2011; Jiang L.-H. et al., 2013). The first is that persistent activation of P2X receptors induces dilation of the small ion-permeating pathway. The second, alternative mechanism is that a separate membrane protein interacts with the P2X receptor and forms the large pores upon activation of P2X receptors. For example, pannexin-1 was shown to form large pores associated with activation of the P2X7 receptor (Pelegrin and Surprenant, 2006).

Two experimental approaches are commonly used to study P2X receptor-dependent large pore formation. The biophysical approach is to patch-clamp record agonist-induced currents with Na^+^ and N-methyl-D-glucamine (NMDG^+^) being the cation in intracellular and extracellular solutions, respectively (Surprenant et al., 1996; Khakh et al., 1999; Virginio et al., 1999a,b). Under such bi-ionic conditions, if the cell membrane is held at a negative potential, activation of P2X receptors induces initially outward currents which decline in amplitude as the receptor activation continues. Within tens of seconds these outwards currents change into inward currents. The current reversal potential exhibits a progressive shift toward the less negative direction (example recordings see Surprenant et al., 1996; Khakh et al., 1999; Virginio et al., 1999a,b; Bo et al., 2003; Jiang et al., 2005). If one assumes the intracellular and extracellular cation concentrations remain unchanged during patch-clamp recording, the shift in the current reversal potential can be interpreted as a result of an increase in the NMDG^+^ permeability of the cell membrane, namely, the open ion channel is poorly permeable to NMDG^+^ at the beginning of receptor activation but significantly increase its NMDG^+^ permeability as the receptor activation continues. Such an interpretation has led to the pore dilation hypothesis (Virginio et al., 1999a; North, 2002). In addition to the P2X receptors, this biophysical approach has been used to show increases in the permeability during activation of other ion channels such as TRPV1 (Chung et al., 2008; Samways et al., 2008; Munns et al., 2015) and TRPA1 (Chen et al., 2009). The second method to study large pore formation is to use fluorescence microscopy or a fluorescence detection system to monitor agonist-induced intracellular accumulation of fluorescence dyes such as YO-PRO-1 and ethidium, or alternatively agonist-induced progressive loss of preloaded fluorescence dyes such as calcein (example recordings see Surprenant et al., 1996; Virginio et al., 1999a,b; Jiang et al., 2005; Sorge et al., 2012). Measurements of dye uptake (or loss) are often made in more physiological solutions containing micromolar concentrations of fluorescent dye and, by and large, avoid the complications associated with complete removal of extracellular physiological cations. One well-documented example of such complications is that the P2X7 and P2X2/5 ion channels activated in extracellular NMDG^+^-containing solutions were somehow trapped in an open state and did not return to the closed state even minutes after agonist application was discontinued (Jiang et al., 2005; Yan et al., 2008; Compan et al., 2012). Therefore, the findings from measurements of fluorescence dye uptake are of much more biological relevance. The amplitude and rate of fluorescence dye uptake are grossly indicative of large pore formation (e.g., Roger et al., 2010; Browne et al., 2013).

It was assumed in previous studies, despite not always being stated explicitly, that P2X receptor-dependent large pores serve as the common pathway permeating NMDG^+^ and fluorescent dye uptake. However, this was challenged in a previous study examining the rat P2X7 receptor heterologously expressed in human embryonic kidney (HEK) 293 cells (Jiang et al., 2005). The study showed that sustained activation of P2X7 receptor in extracellular Na^+^-containing solutions induced robust YO-PRO-1 dye uptake but, surprisingly, no increase in the P_NMDG_/P_Na_. In addition, the study found that removal of a cysteine-rich microdomain in the proximal part of the intracellular C-terminus almost completely abolished agonist-induced reversal potential shift under bi-ionic conditions without compromising agonist-induced YO-PRO-1 uptake. In fact, as compared to the wild-type receptor, expression of the deletion mutant receptor resulted in higher YO-PRO-1 uptake in both Na^+^-containing and NMDG^+^-containing solutions (Jiang et al., 2005). These two independent lines of evidence strongly argue against the idea that a same molecular mechanism is used to mediate the entry of both NMDG^+^ and YO-PRO-1 into the cell. In HEK293 cells heterologously expressing the rat P2X2 receptor, a recent study has found that ATP activation of the P2X2 receptor in extracellular Na^+^-containing solutions induced no increase in the P_NMDG_/P_Na_ (Li et al., 2015). The study has elegantly introduced a reservoir model to support the notion that the reversal potential shift simply arises from substantial reduction in the intracellular Na^+^ concentration and increase in the intracellular NMDG^+^ concentration during prolonged P2X2 ion channel opening. In their model, the P2X2 ion channel is NMDG^+^-permeable, albeit with the P_NMDG_/P_Na_ of 0.05, but there is no need for an increase in the NMDG^+^ permeability, in other words, no pore dilation! The study has demonstrated that the open P2X2 ion channel permeates NMDG^+^ as quickly as small cations like Na^+^, but not as easily as the latter ions. The P2X7 open ion channels also exhibit extremely low, if any, NMDG^+^ permeability (P_NMDG_/P_Na_ ~ 0.03-0.04; Virginio et al., 1999a; Jiang et al., 2005). Structural modeling based on the open state structure of zebrafish P2X4 receptor (Hattori and Gouaux, 2012) positions the Cα atoms of three S342 residues in the physical gate of the ion-permeating pathway as being 6.4 Å from the central axis in both rat and human P2X7 receptors (Browne et al., 2013; Jiang L.-H. et al., 2013; Figure 1D). NMDG^+^ has a size of 6 Å × 6 Å × 12.5Å and therefore, as proposed in a recent study (Browne et al., 2013), the P2X7 open ion channels may be sufficiently wide to permeate NMDG^+^.

The commonly used fluorescent dyes are, however, considerably larger in size than NMDG^+^, for example YO-PRO-1 (7 Å × 8 Å × 19Å) and ethidium (6.5 Å × 11 Å × 13Å) (Browne et al., 2013). How do the fluorescent dyes come across the cell membrane, also through the ion-permeating pathway? Previous studies showed YO-PRO-1 uptake following activation of P2X2, P2X2/3, and P2X4 receptors (Khakh et al., 1999; Virginio et al., 1999b). The open ion channels of these receptors, if permitting passage of YO-PRO-1, have to open much more widely than the above-mentioned ion-permeating pathway revealed in the open state structure of zebrafish P2X4 receptor (Hattori and Gouaux, 2012). Such a possibility remains to be tested. A recent study has investigated whether the rat P2X7 ion channel was able to permeate large molecules, including the cationic fluorescent dyes YO-PRO-1 and ethidium, the anionic fluorescent dye fluorescein isothiocyanate (FITC; 8.5 Å × 11 Å × 14.5Å), and neutral cysteine-modifying 2-aminoethyl methanethiosulfonate (MTSEA; 5 Å × 5 Å × 10Å), MTSEA-biotin (7.5 Å × 8 Å × 18.5Å) and MTS-rhodamine (9 Å × 14 Å × 16.5 Å) (Browne et al., 2013). ATP-induced ionic currents and YO-PRO-1 uptake both strongly depend on membrane potential, the driving force for movement of charged molecules. ATP also induced uptake of ethidium and FITC in a correlating fashion even though these two dyes bear opposite charges. ATP-induced ethidium uptake was reduced and by contrast ATP-induced FITC uptake was increased by membrane depolarization. Furthermore, introduction of a positive charge by T348K mutation or neutralization of a negative charge by D352N in the small ion-permeating pathway resulted in a decrease in ATP-induced ethidium uptake but an increase in ATP-induced FITC uptake. Finally, MTSEA-biotin and MTS-rhodamine as well as MTSEA readily modified cysteine replacing G345, a position internal to the narrowest part of the P2X7 open ion channel, and inhibited ATP-induced ionic currents and ethidium uptake. These results provide direct evidence to demonstrate that the rat P2X7 open ion channel can permeate large molecules. However, to accomplish this, the open ion channels need to be a minimum of 14 Å wide. This is noticeably wider than the ion-permeating pathway in the open state models of rat and human P2X7 receptors, supporting the notion that the open ion channel dilates (Virginio et al., 1999a; Browne et al., 2013). Structural determination of the ion-permeating pathway of a mammalian P2X receptor in the open state will provide the key answer to whether or how the open ion channel allows passage of fluorescent dyes.

In parallel with these efforts to understand P2X receptor-dependent large pore formation, studies have accumulated evidence to show the importance of such receptor functionality. For example, P2X7 receptor-dependent large pore formation has been identified as a crucial factor associated with disease conditions such as chronic pain (Sorge et al., 2012), osteoporosis (Syberg et al., 2012) and geographic atrophy (Fowler et al., 2014). Furthermore, preferential inhibition of P2X7 receptor-dependent large pore formation has been proposed in a recent study to be the molecular mechanism underpinning the anti-inflammatory activity of nucleoside reverse transcriptase inhibitors, a class of clinically proven drugs treating HIV (Fowler et al., 2014). Selective targeting of P2X7 receptor-dependent large pore formation appears a promising and novel pharmacological intervention (Jiang, 2015). Therefore, it becomes increasingly interesting and important to gain a better mechanistic insight into large pore formation after activation of P2X receptors, in particular P2X7 receptors.

## Author contributions

LW, L-HJ and DL led the discussion; EC contributed to the discussion and generated the structural models. L-HJ wrote the manuscript, and all authors commented the manuscript.

### Conflict of interest statement

The authors declare that the research was conducted in the absence of any commercial or financial relationships that could be construed as a potential conflict of interest.
